# Development of an UPLC-MS/MS method for quantitative analysis of abexinostat levels in rat plasma and application of pharmacokinetics

**DOI:** 10.1186/s13065-024-01144-z

**Published:** 2024-02-20

**Authors:** Yige Yu, Jinyu Hu, Xiaohai Chen, Hua-lu Wu, Anzhou Wang, Congrong Tang

**Affiliations:** Department of Pharmacy, the First Affiliated Hospital of Wenzhou Medical University, Wenzhou, 325000 China

**Keywords:** Abexinostat, Pharmacokinetics, UPLC-MS/MS, Givinostat

## Abstract

Broad-spectrum histone deacetylase inhibitors (HDACi) have excellent anti-tumor effects, such as abexinostat, which was a novel oral HDACi that was widely used in clinical treatment. The purpose of this study was to establish a rapid and reliable method for the detection of abexinostat concentrations in rat plasma using ultra-performance liquid chromatography tandem mass spectrometry (UPLC-MS/MS). The mobile phase we used was acetonitrile and 0.1% formic acid, and the internal standard (IS) was givinostat. Selective reaction monitoring (SRM) was used for detection with ion transitions at *m/z* 397.93 → 200.19 for abexinostat and *m/z* 422.01 → 186.11 for givinostat, respectively. The intra-day and inter-day precision of abexinostat were less than 11.5% and the intra-day and inter-day accuracy ranged from − 10.7% to 9.7% using this method. During the analysis process, the stability of the test sample was reliable. In addition, the recovery and matrix effects of this method were within acceptable limits. Finally, the method presented in this paper enabled accurate and quick determination of abexinostat levels in rat plasma from the pharmacokinetic study following gavage at a dose of 8.0 mg/kg abexinostat.

## Introduction

A balanced ratio of histone deacetylase and histone acetylase expression in malignant tissues results in the expression of a malignant phenotype. Histone deacetylase inhibitors (HDACi) retains acetyl groups on histones by inhibiting deacetylated proteins, which may favor apoptotic genes in tumor cells [1]. The biological effects of HDACi include inhibiting cell proliferation, promoting cell differentiation, and apoptosis in tumor cells [2, 3]. Previous studies have shown that increased abexinostat concentration promoted apoptosis in Hodgkin lymphoma cell lines and regulated the apoptosis process through the NF-κB signaling pathway [4, 5]. This kind of broad-spectrum HDACi currently approved for clinical research has multiple targets and has a certain anti-tumor effect [6].

**Fig. 1:**
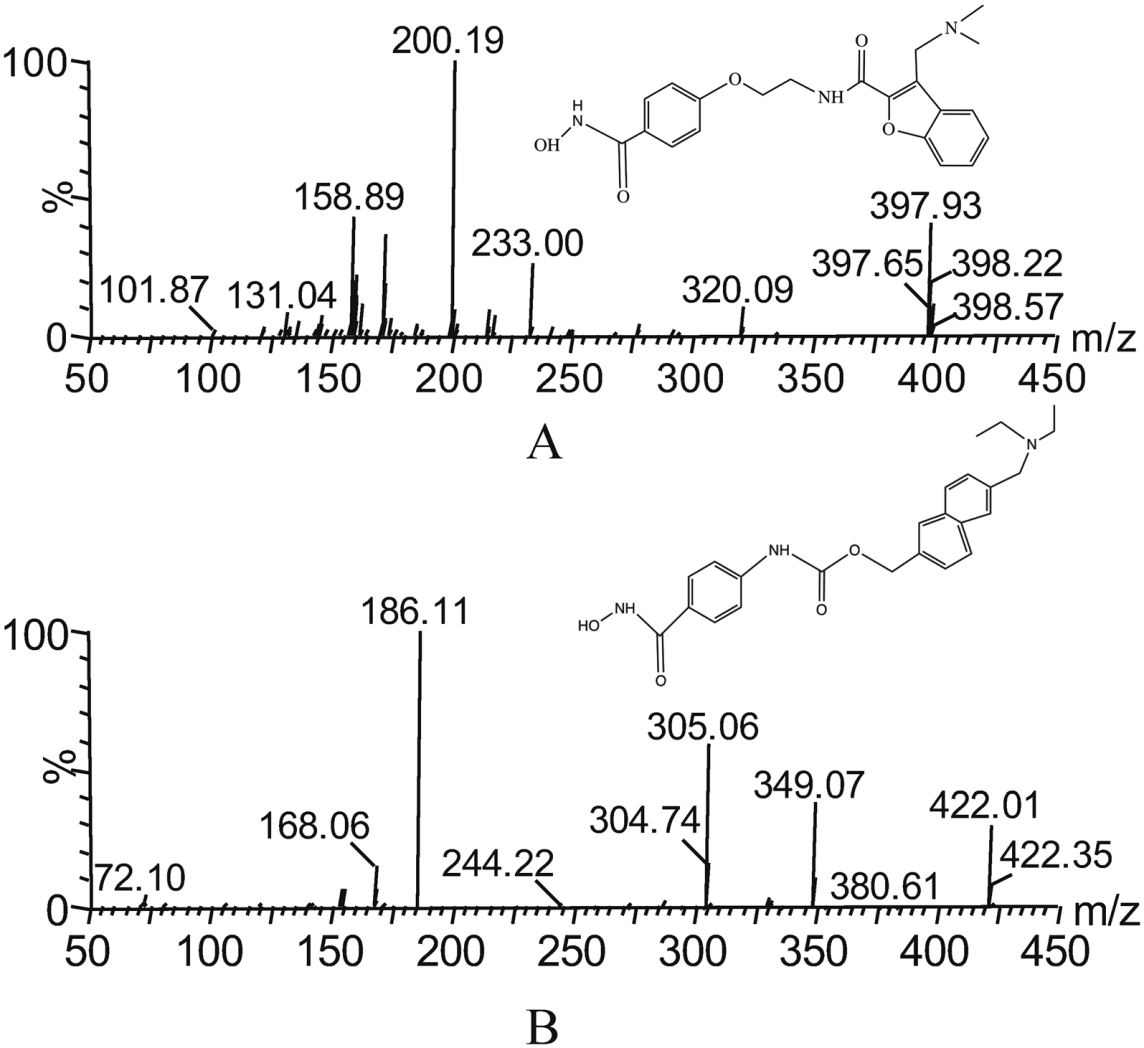
Mass spectra of abexinostat (**A**) and givinostat (**B**) in this study

Abexinostat (Fig. 1A), a new broad-spectrum HDACi, has therapeutic differences and very limited benefit for patients with different types of tumors treated alone [7]. In Phase I preclinical studies of various types of leukemia with abexinostat, only 1 out of 12 evaluable patients had stable disease [8]. The most common grade 3 treatment-related adverse events were thrombocytopenia (29%) and neutropenia (24%). These hematological adverse symptoms are mainly related to the persistent toxic effects of HDACi and the unclear mechanism of action. In a phase II non-Hodgkin lymphoma patient study, abexinostat demonstrated durable tumor control in patients with follicular lymphoma (FL), T-cell lymphoma (TCL), diffuse large B-cell lymphoma (DLBCL) [9]. The drug was well tolerated during administration and the use of lower doses could reduce hematological toxicity [5]. The number of research samples in the above-mentioned preliminary research is very limited, and statistical research should be carried out on a larger population base in the subsequent research. The toxicity study of abexinostat shows that its toxicity is controllable, and it has great prospects for the future combination therapy for tumor diseases.

Until now, none of the current studies mentioned the use of UPLC-MS/MS to detect abexinostat concentrations in plasma. In a phase II clinical maximum toxicity tolerance experiment, it was found that when patients took 30, 45, and 60 mg/m^2^, respectively, 2/8 patients in the 60 mg/m^2^ group were found to have dose-limited toxicities (DLTs) (grade 5 acute renal failure, grade 3 long-term diarrhea) [10]. This indicated that with an increase in the plasma concentration of abexinostat, the frequency of adverse reactions increased. In another clinical trial, when treating metastatic sarcoma with abexinostat and doxorubicin, DLTs were also used to determine the maximum tolerability of patients taking abexinostat. The research did not report on detecting the concentration of abexinostat in plasma. However, it is important to measure the level of drugs in plasma when studying drug-drug interactions. [11]. In this study, we established a method to measure the level of abexinostat in plasma to provide a scientific basis for the clinical application such as the drug-drug interaction affecting the plasma level of the drug in combination with abexinostat. Here we established an effective UPLC-MS/MS methodology that meets the requirements in vivo pharmacokinetic experiments. This methodology will provide an effective solution for clinical detection of abexinostat concentrations in plasma.

## Experimental

### Chemical reagent

Abexinostat (Fig. 1A) and givinostat (used as internal standard, IS, Fig. 1B) were provided by Shanghai Chuangsai Technology Co., Ltd. (Shanghai, China) with > 98% purity. The methanol and acetonitrile used in the experimental procedure were both HPLC grade, provided by Merck Company (Darm-stadt, Germany). Formic acid provided by Anaqua Chemicals Supply (ACS, American) also was HPLC grade. Milli-Q pure water system was used to prepare ultrapure water (Millipore, Bedford, USA).

### Animal experiments

Animals (weight of 200 g ± 20 g) were purchased from The First Affiliated Hospital of Wenzhou Medical University (Zhejiang, China). All animals were acclimated to the standard environment of laboratory animals for 1 week before the experiment. The experimental operations were in compliance with the standards of the Institutional Ethics Committee of the First Affiliated Hospital of Wenzhou Medical University (Zhejiang, China) [12, 13].

### Instrument and parameter conditions

Quantitative analysis was performed using a UPLC-MS/MS system consisting of a Waters Xevo TQ-S triple quadrupole tandem mass spectrometer and a Waters ACQUITY UPLC I-Class system (Milford, MA, USA) used in this experiment. Accurate molecular weight calculations were achieved using an electrospray ionization (ESI) source (Milford, MA, USA) with ultra-high sensitivity. The system was chromatographed on an Acquity BEH C18 column (2.1 mm × 50 mm, 1.7 μm; Milford, MA, USA) at 40 °C with acetonitrile and 0.1% formic acid as mobile phases. Gradient elution was as follows: 0 − 0.5 min (acetonitrile, 10%) and 0.5–1.0 min (acetonitrile, 10–90%), 1.0–1.4 min (acetonitrile, 90%) and 1.4–1.5 min (acetonitrile, 90–10%) at 0.40 mL/min. Finally, the mobile phase was maintained 10% acetonitrile between 1.5 and 2.0 min for equilibration. A 2.0 μL sample was drawn from each vial as the injection volume, and the entire analysis process time was 2.0 min. In UPLC-MS/MS, we used positive ion mode to detect abexinostat and IS with ion transition of *m/z* 397.93 → 200.19 and 422.01 → 186.11, respectively. 25 eV and 30 V were the collision energy and cone voltage for both.

### Standards, standard curves and quality controls

For the preparation of control and standard curves, we dissolved abexinostat and IS in methanol at a concentration of 1.00 mg/mL. Both the abexinostat working solution and the 100 ng/mL IS working solution were diluted with methanol from their respective stock solutions. The corresponding working solution (10 μL) was diluted tenfold and operated by adding rat blank plasma (90 μL) to obtain a calibration curve with a concentration range of 2.00–100 ng/mL. The concentrations at each point in the standard curve were set to 2.00, 5.00, 10, 20, 50, 100 ng/mL, respectively. Final concentrations of QC samples were 2.00 (lower limit of quantitation, LLOQ), 5.00 (low quality control, LQC), 40 (median quality control, MQC), and 80 (high quality control, HQC) ng/mL.

### Sample preparation

Plasma protein composition interfered with detection sensitivity. The protein precipitation method in this study was to add acetonitrile to the plasma, and the protein was fully precipitated and then centrifuged. We added 100 μL of the plasma sample along with 10 μL of the IS working solution (concentration of 100 ng/mL) in an Eppendorf (EP) tube. Mixing well and adding acetonitrile at a ratio of 1:3 to precipitate the protein in the EP tube, and centrifuged at 13,000 rpm for 10 min at 4 °C. 100 μL of the supernatant was transferred to an autosampler vial for quantitative analysis by the UPLC-MS/MS system.

### Method validation

The principles of the analytical methods in this study were performed in accordance with the FDA’s “Guidance for Industry: Bioanalytical Method Validation” [14], including calibration curves, selectivity, LLOQ, recovery, matrix effects, precision and accuracy, and stability.

### Pharmacokinetic studies

Sprague–Dawley Rats Were Provided By The First Affiliated Hospital Of Wenzhou Medical University (Zhejiang, China), And The Animal Study Was Reviewed And Approved By Ethics Committee Of The First Affiliated Hospital of Wenzhou Medical University (WYYY-IACUC-AEC-2023-055). All rats were fasted for 24 h before the experimental animals, and a single gavage dose of 8.00 mg/kg of abexinostat was administered to 6 rats after fasting. At 20 and 40 min, 1, 1.5, 2, 3, 4, 6, 8, 12, 24, and 48 h after gavage, 300 μL of whole blood was collected from the tail vein of the rat and put into heparinized tubes. Then, the blood sample was centrifuged at 8000 rpm for 5 min. 100 μL of plasma was obtained and frozen at − 80 °C for further processing and analysis.

Animals were euthanized using the anesthesia method according to the AVMA Guidelines for Animal Euthanasia. All experimental animals were euthanized with intravenous pentobarbital (150 mg/kg) after completion of the experiment. After ensuring that the animals were free of life pointers, they were packaged and cremated.

The main parameters of pharmacokinetics were analyzed by Drug and Statistics (DAS, Version 3.0 software, Shanghai University of Traditional Chinese Medicine, China).

## Results and discussion

### Method development

**Fig. 2:**
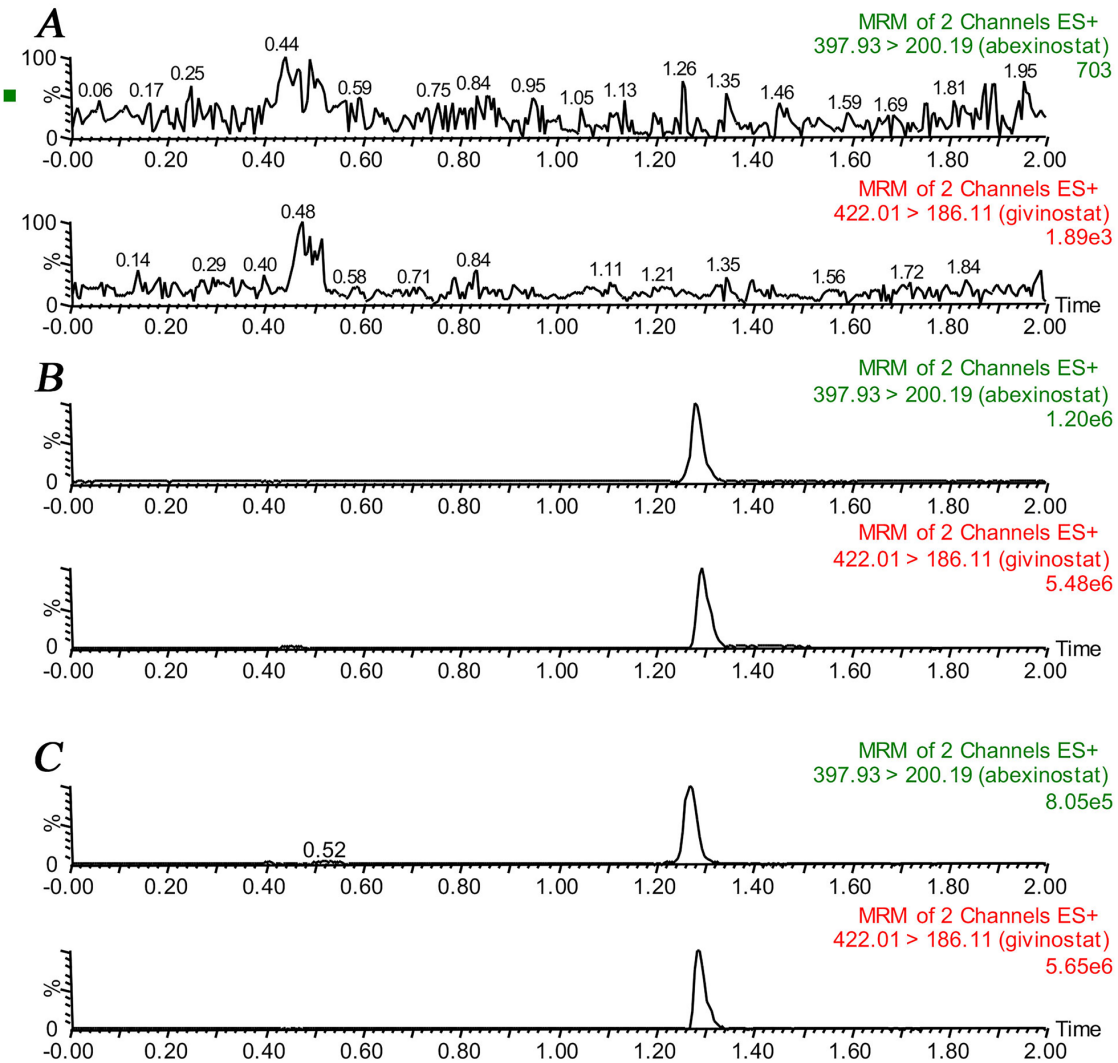
Representative chromatograms of abexinostat and IS in rat plasma: **A** blank plasma; **B** blank plasma spiked with analyte at LLOQ and IS; **C** plasma sample collected from a rat at 40 min after intragastric administration of 8.00 mg/kg abexinostat

In general, the separation of the analytes is closely related to chromatographic conditions such as mobile phase composition and choice of solid phase materials (15, 16). In this study, the chromatographic separation conditions were screened and finally determined as mobile phase gradient elution with acetonitrile and 0.1% formic acid, and the chromatographic column was an Acquity BEH C18 column (2.1 mm x 50 mm, 1.7 μm). In order to refine the chromatographic conditions, optimization of parameter settings in mass spectrometers is essential. To obtain the highest fragmentation intensity, the mass spectrometry parameters were optimized and the collision-induced dissociation energy was increased, because different collision voltages have a great influence on the imaging results. Finally, we chose the collision energy of 25 eV and the cone voltage of 30 V for both abexinostat and IS. Although at more suitable collision energies, the ions still had some potential fragment ions during the fragmentation process. We chose abexinostat and IS with ion transitions of *m/z* 397.93 → 200.19 and 422.01 → 186.11, respectively, because of the lower noise levels and high sensitivity. Under optimal separation conditions, the retention times of abexinostat and IS were 1.28 min and 1.30 min, respectively (Fig. 2). From the results, it could be concluded that the lack of interference in the blank plasma supports the specificity of the method. In addition, we speculated that different injection volumes may have an impact on the detection results when detecting the LLOQ, and finally we selected 2.0 μL as the method of injection volume.

Under the experimental conditions identified above, another advantage of this study was using UPLC-MS/MS for the first time which required short run time (2.0 min), provided accurate and cost-effective analysis of abexinostat using very low sample volume. In the pharmacokinetic study, the characteristics of short time and high accuracy brought great convenience to the determination of abexinostat level in plasma.

### Method validation

#### Specificity

Detection of abexinostat and IS in rat plasma was conducted using UPLC-MS/MS under optimized conditions with retention times of 1.28 min and 1.30 min, respectively. By comparing 6 samples from different rats and blank plasma plus abexinostat at the LLOQ concentration, in addition to sample from real rat plus IS, no potential interfering substances were found after analysis (Fig. 2). Therefore, this method had a relatively good selectivity.

#### Calibration and LLOQ

The calibration curve of abexinostat in rat plasma was linear, and the obtained representative linear regression equation was Y = 0.157372X − 0.00682589 (*r*^2^ = 0.999) (Y was the peak area ratio, X was the corresponding concentration). The LLOQ of 2.00 ng/ml indicated the detection sensitivity of the method, and its precision and accuracy were 11.5% and − 10.7%, respectively.

#### Accuracy and precision

The precision and accuracy of the method were calculated by testing QC samples over three days at three different concentration levels (n = 6) of 5.00, 40 and 80 ng/mL. As listed in Table 1, the intra-day and inter-day accuracies of abexinostat were lower than 9.7%, and the precision was lower than 10.8% that at the three identified QC concentration levels. Therefore, the precision and accuracy of the described UPLC-MS/MS method for the quantitative analysis of abexinostat in rat plasma met the requirements.

**Table 1:** The precision and accuracy of abexinostat in rat plasma (n = 6)

Analyte	Concentration (ng/mL)	Intra-day		Inter-day	
		RSD %	RE %	RSD %	RE %
	2.00	3.4	0.9	11.5	− 10.7
Abexinostat	5.00	10.8	–2.2	10.5	9.7
	40	5.4	–0.7	9.8	0.9
	80	2.9	3.1	6.8	2.2

#### Recovery and matrix effect

In this study, recovery and matrix effects were calculated using IS normalization, which was determined by the peak areas of the analyte and IS:

Recovery = peak area of extract sample/ peak area of blank extract spiked with neat solution.

Matrix effect = peak area of blank extract spiked with neat solution / peak area of neat solution.

At three different concentrations of 5.00, 40, and 80 ng/mL, the mean recovery of abexinostat ranged from 81.3 to 99.6%, and the mean range of matrix effect of abexinostat was from 110.2% to 111.8% (Table 2). These data demonstrated that abexinostat had no significant matrix effect measured in rat plasma under optimized UPLC-MS/MS conditions.

**Table 2:** Recovery and matrix effect of abexinostat in rat plasma (n = 6)

Analyte	Concentration (ng/mL)	Recovery (%)		Matrix effect (%)	
		Mean ± SD	RSD (%)	Mean ± SD	RSD (%)
	5.00	81.3 ± 11.3	13.8	111.5 ± 13.6	11.4
Abexinostat	40	96.1 ± 2.0	2.1	110.2 ± 2.9	2.6
	80	99.6 ± 3.3	3.3	111.8 ± 4.3	4.5

#### Stability

Stability experiments to test abexinostat in plasma under different storage and handling conditions were done to investigate whether abexinostat was still stable in rat plasma. The stability test results were in Table 3. We found that plasma samples containing abexinostat were stable after three cycles of full freezing (− 80 °C)/thawing (room temperature) and at − 80 °C for at least 3 weeks. Furthermore, the assay remained stable in the autosampler (10 °C) for 4 h and 3 h at room temperature. Relative errors (RE) within ± 15% were considered stable by data comparison.

**Table 3:** Stability results of abexinostat in plasma under different conditions (n = 5)

Analyte	Concentration (ng/mL)	Room temperature, 3 h		Autosampler 10 ℃, 4 h		Three freeze–thaw		− 80 ℃, 3 weeks	
		RSD (%)	RE (%)	RSD (%)	RE (%)	RSD (%)	RE (%)	RSD (%)	RE (%)
Abexinostat	5.00	4.6	− 5.1	10.6	− 1.7	7.9	− 7.3	6.3	5.4
	40	3.4	2.2	5.5	15.0	2.2	− 10.3	6.9	3.3
	80	3.7	0.2	3.1	− 11.9	2.5	13.8	4.1	− 1.6

#### Pharmacokinetics study

**Fig. 3:**
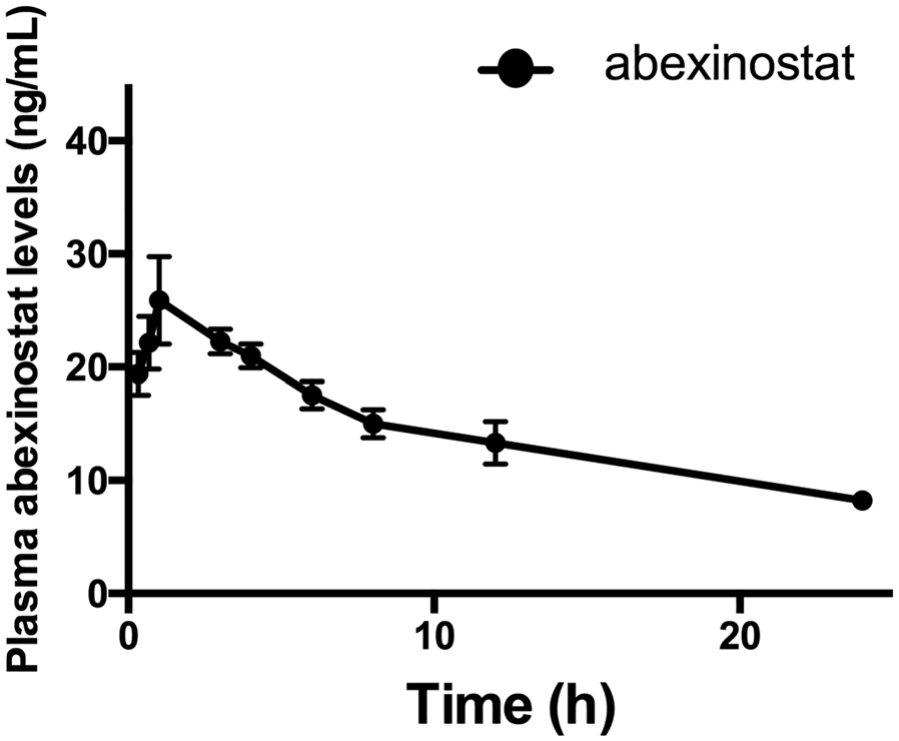
Mean plasma concentration–time curves of abexinostat in rats after intragastric administration of abexinostat at a single dose of 8.00 mg/kg. (n = 6)

After oral administration of abexinostat to rats, the absorption was rapid, and the maximum plasma concentration (C_max_) was 28.20 ± 7.67 ng/mL (Table 4). In addition, the time to peak (T_max_) in rat plasma was about 1.39 ± 1.29 h, and it also can be estimated from Fig. 3 that the plasma concentration peaks around 1 h after oral administration of abexinostat. Furthermore, the half-life (t_1/2_) of abexinostat in rats was 13.80 ± 2.50 h. In the present study, the pharmacokinetic study of abexinostat in rats by UPLC-MS/MS is reported for the first time. Considering that our study was conducted in rats, and there are large differences between humans and animals, so more research should be done to explore the pharmacokinetics of abexinostat in humans.

**Table 4:** The main pharmacokinetic parameters of abexinostat in rat plasma after intragastric administration of abexinostat at a single dose of 8.00 mg/kg

Parameter	Abexinostat
AUC_0-t_ (ng/mL*h)	460.57 ± 38.89
AUC_0-∞ (_ng/mL*h)	501.25 ± 40.15
MRT_0-t_ (h)	14.76 ± 0.80
MRT_0-∞_ (h)	19.14 ± 2.55
t_1/2_ (h)	13.80 ± 2.50
T_max_ (h)	1.39 ± 1.29
CL_z_/F (L/h/kg)	16.04 ± 1.26
C_max_ (ng/mL)	28.20 ± 7.67

(n = 6, Mean ± SD)

## Conclusions

In conclusion, a robust, fast and reliable UPLC-MS/MS method for the quantification of abexinostat in rat plasma was developed and validated. This optimized method has the distinct advantages of a short analysis time (2.0 min) and stable assay conditions. The level of abexinostat in plasma was detected with certain accuracy under different severe storage conditions. The data of the oral dose of 8.00 mg/kg abexinostat in pharmacokinetic studies in rats indicated that this UPLC-MS/MS optimization method was feasible for future application in pharmacokinetic studies.

## Data Availability

The raw data supporting the conclusion of this article will be made available by the authors, without undue reservation.
